# Chronotype predicts working memory-dependent regional cerebral oxygenation under conditions of normal sleep and following a single night of sleep extension

**DOI:** 10.1038/s41598-023-45238-5

**Published:** 2023-10-19

**Authors:** Joaquin U. Gonzales, Jacob R. Dellinger, Cayla Clark

**Affiliations:** Department of Kinesiology and Sport Management, Texas Tech University, Box 43011, Lubbock, TX 79409-3011 USA

**Keywords:** Neurology, Cognitive control

## Abstract

The aim of this study was to test the hypothesis that the association between sleep duration and brain activation as assessed by regional cerebral oxygenation using near-infrared spectroscopy (NIRS) is dependent on chronotype. Sleep was tracked across two weeks by actigraphy in 22 adults instructed to keep their normal sleep behavior. Chronotype was assessed by the midpoint of sleep on free days corrected for sleep debt on workdays (MSFsc). Prefrontal cerebral oxygenation (ΔHbDiff) during a visuospatial working memory task was measured in the morning after a night of normal sleep and after one night of extended sleep. Sleep extension was included to experimentally test the robustness of the association between sleep duration and ΔHbDiff. Habitual sleep duration (r = 0.43, *p* = 0.04) and MSFsc (r = − 0.66, *p* < 0.001) were significantly correlated with ΔHbDiff. After adjusting for MSFsc the relationship between sleep duration and ΔHbDiff was reduced to nonsignificant levels (r = 0.34, *p* = 0.11), while adjusting for sleep duration did not change the significant relationship between MSFsc and ΔHbDiff (r = − 0.62, *p* = 0.001). One night of sleep extension increased sleep duration by 140 min, on average, but no change in ΔHbDiff was observed. Dividing participants into earlier and later chronotypes revealed greater ΔHbDiff responses in earlier chronotypes that persisted after the night of sleep extension (mean ΔHbDiff difference = 1.35 μM, t = 2.87, *p* = 0.006, Hedges’ *g* = 0.89). These results find chronotype to predict regional cerebral oxygenation responses during working memory processing under conditions of normal sleep and following a single night of sleep extension.

## Introduction

Sleep loss represents a challenge for the brain to meet neural processing demands. For instance, one night of sleep deprivation increases brain activation in the prefrontal cortex during tasks of attention and working memory^1,2^. The elevation in prefrontal activation after sleep loss is hypothesized to represent a functional compensation to maintain cognitive performance in the midst of impaired neural efficiency and/or decreased neural activity in other parts of the brain^3^. This sleep-related alteration in prefrontal activation has been demonstrated using various technologies that measure cerebral oxygenation including functional magnetic resonance imaging and near-infrared spectroscopy (NIRS)^4^. Cerebral oxygenation is considered a proxy measure for brain activation since neural activity is accompanied by regional changes in cerebral blood flow for the delivery and use of oxygen. While it is well known that sleep loss resulting from sleep restriction or deprivation elevates prefrontal activation during cognitive challenges^3^, less is understood about the influence of longer sleep durations.

Few studies have examined the influence of longer sleep duration on brain activation during cognitive functioning. Cross-sectional studies find sleep duration the night prior to testing is positively correlated with prefrontal cerebral oxygenation responses to verbal fluency^5^ and working memory tasks^6^. These studies suggest that longer sleep durations are associated with elevated brain activation during cognitive tasks. Our laboratory recently demonstrated in an experimental study that five nights of extended sleep increases prefrontal cerebral oxygenation responses to various tests that depend on attention, spatial rotation ability, and working memory without an accompanying improvement in cognitive performance^7^. This finding suggest that longer sleep durations, like sleep loss, may elevate prefrontal activation to maintain cognitive performance.

A shortcoming of our previous work^7^ along with others^5^ is that attention was not paid to the chronotype of the individual. Chronotype refers to when an individual’s endogenous circadian clock synchronizes to the 24-h light–dark cycle^8^. In accordance with the individual’s circadian preference, some have a propensity for an earlier bedtime and wake-up time and wake at an earlier circadian phase^9,10^, while others prefer a later bedtime and wake-up time. Chronotype is a sleep characteristic that has been shown to influence brain activation during cognitive functioning. For instance, early chronotypes are reported to have higher activation in the middle frontal gyrus than late chronotypes when performing a working memory N-back task in the morning, while late chronotypes have higher activation in the thalamus for the same task when performed in the evening^11^. While sleep duration on weekdays may not significantly differ between early and late chronotypes^12^, late chronotypes are subjected to less time in bed and less sleep duration than their sleep need during weekdays^10^ which may alter brain activation patterns. Whether chronotype contributes to or explains the association between longer sleep duration and higher cerebral oxygenation during cognitive functioning is currently unclear.

The aim of the present study was to test the hypothesis that the association between sleep duration and brain activation during a working memory task as assessed by prefrontal cerebral oxygenation using NIRS is dependent on chronotype. Furthermore, we hypothesized that chronotype rather than sleep duration would impact the change in prefrontal cerebral oxygenation during a working memory task following a single night of sleep extension.

## Methods

### Participants

Twenty-two adults (16 women, 6 men) with an average age of 60 ± 15y, height of 166 ± 7 cm, weight of 70 ± 16 kg, and body mass index of 25.6 kg/m^2^ completed this study. All participants provided written informed consent prior to screening and testing. The Human Research Protection Program at Texas Tech University provided ethical approval for this study (#IRB2020-896), and the study procedures conformed to standards set by the Declaration of Helsinki. Participants were included if they were ≥ 40 years and considered themselves to have regular sleep habits based on consistent bedtime and wake times. Participants were excluded if they showed signs of insomnia using the Insomnia Symptom Questionnaire^13^, if they reported to have sleep apnea, took pills to improve sleep, or had a personal history of stroke, myocardial infarction, or diabetes mellitus.

As the primary objective of this study was to examine the influence of chronotype on the relationship between sleep duration and brain activation, we used results from past research that reported sleep duration to associate with prefrontal cerebral oxygenation responses to a working memory task in order to determine the necessary sample size. Yeung et al.^6^ reported a significant positive correlation between sleep duration and prefrontal oxy-hemoglobin levels in the left hemisphere (r = 0.53, coefficient of determination r^2^ = 0.28). Using an alpha of 0.05 and a desired power of 0.80, G*Power (version 3.1.9.4) calculated a sample size of 18 participants was needed to achieve an estimated effect size of 0.53 using a one-tailed distribution.

### Study design

**Figure 1:**
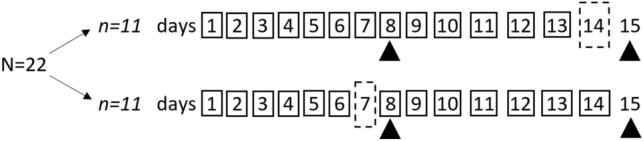
Participants extended sleep (dashed rectangle) the night prior to testing on the 7th or 14th night within a two week period. Participants were instructed to keep their usual sleep schedule (solid boxes) for the other nights. Cerebral oxygenation was measured during a working memory task (solid triangles) at the same time each morning on the 8th and 15th day after an overnight fasting period.

At the first visit, participants were familiarized with the cognitive function test while wearing the NIRS device, given instructions about wearing the accelerometer, and asked to maintain their habitual sleep habits during the study period. Sleep was then monitored across 14 consecutive nights. Participants arrived in the morning after the 7th night (n = 11) and 14th night (n = 11) with half of the participants extending sleep on the 7th night of sleep and the other half extending sleep on the 14th night (see Fig. 1). To extend sleep, participants were asked to spend at least 10 h in bed trying to sleep consistent with our past work^7^. At each study visit, participants completed the cognitive function test while wearing the NIRS device. Only in the morning after the night of normal sleep, participants were also asked to complete the Epworth Sleepiness Scale^14^ to assess sleepiness. All study visits took place in the morning after an overnight fast to avoid the influence of dietary compounds (e.g., caffeine) on arousal. Time for each visit was kept constant between visits for each participant, which required participants to extend sleep by going to bed earlier rather than sleeping later. All participants scheduled their study visits between 7:00 and 9:00 a.m., with the exception of one participant that had an 11:00 a.m. visit time.

### Sleep monitoring

A triaxial accelerometer (GT9X, ActiGraph, Pensacola, FL, USA) was worn by participants on their non-dominant wrist with instructions only to remove the accelerometer during bathing or swimming. ActiLife’s sleep analysis software using a combination of Tudor-Locke and Cole-Kripke algorithms was used to identify sleep periods. Sleep periods were manually adjusted, if needed, by researchers from sleep diaries of sleep/wake cycles logged by participants. Variables of interest from the sleep analysis included time in bed, sleep duration, wake after sleep onset (WASO), nocturnal awakenings, and sleep efficiency (sleep duration/total time in bed), and sleep fragmentation index. Sleep fragmentation index was calculated by ActiLife software by summing movement (total of scored awake minutes divided by the total time in bed) and fragmentation indices (percentage of 1-min periods of sleep versus all periods of sleep). A 12-night average was calculated to reflect habitual sleep. The average excluded the 7th and 14th night for all participants in case the night prior to testing altered sleep behavior or quality.

Chronotype was assessed by calculation of the midpoint of sleep on free days (MSF) corrected for sleep debt on workdays (MSFsc)^8^. This proxy measurement for chronotype is strongly correlated with delayed dim light melatonin onset^15^, which is considered the gold standard measurement of human circadian timing. In addition to chronotype, social jet lag was estimated due to its potential of being a significant covariate in our investigation. Social jet lag is defined as the difference between unconstrained and constrained sleep times, and in this study was calculated as the difference in the midpoint of sleep on workdays (MSW) and on free days (MSF), hence MSF—MSW in minutes.

### Cerebral response to cognitive challenge

A NIRS device (PortaLite, Artinis Medical Systems, The Netherlands) was used to measure regional cerebral oxygenation in the prefrontal cortex during a delayed, nonverbal, matching to sample task that relies heavily on visuospatial working memory^16^. This test was chosen as working memory is a common cognitive domain recruited by many different cognitive challenges used by previous studies^1,3,6,17,18^, and the prefrontal cortex is a neural network relied upon heavily during matching to sample tasks^19^. Participants viewed a 4 × 4 red and white block design, were given a 5-s delay, and then shown two designs to select the original design. A visual example of the matching to sample test can be viewed elsewhere^20^. Twenty trials of this cognitive task was completed on a computer using Automated Neuropsychological Assessment Metrics software (VistaLife Sciences, Parker CO). A small NIRS device was placed on the left side of the forehead, about 2 cm from the midline above the eyebrow, and was held in place with a headband. The device was covered with a small black cloth in order to minimize light interference. Near-infrared light was transmitted with wavelengths of 760 and 850 nm by a light-emitting diode and was received by a photodiode at 35 mm interoptode distance. Changes in oxygenated and deoxygenated hemoglobin was measured at 25 Hz by the NIRS device using a modified Lambert–Beer law algorithm. An age-dependent path length factor was calculated using NIRS software^21^. The primary variable of interest was the difference in oxygenated and deoxygenated hemoglobin (HbDiff) which reflects cerebral oxygenation^22^. HbDiff was averaged across the entire cognitive test and expressed as a change from rest (ΔHbDiff). In terms of cognitive performance, average reaction time and throughput score (correct responses per minute of available response time) from the matching to sample task were recorded.

### Statistical analysis

Pearson correlations coefficients were used to report relationships between variables, and partial regression was used to control for variance explained by chronotype. Two-tailed paired t-tests were used to compare variables between the habitual sleep and the night of extended sleep. A two-way ANOVA with Holm-Sidak posthoc testing was used to compare prefrontal cerebral oxygenation between chronotype groups across habitual and extended sleep conditions. Lastly, the Hedges’ *g* statistic was calculated for effect sizes and can be interpreted as small (≥ 0.20), medium (≥ 0.50), or large (≥ 0.80). Statistical significance was set a priori at *p* < 0.05, and values are reported as mean ± SD.

## Results

### Association between habitual sleep duration and prefrontal oxygenation

The ΔHbDiff (i.e., cerebral oxygenation response) increased during the working memory task by an average of 2.18 ± 1.59 μM. Only one participant failed to show an increase in cerebral oxygenation during the working memory task relative to rest. There was no difference in ΔHbDiff between participants completing the task for their second (1.89 ± 1.39 μM) or third time (2.47 ± 1.79 μM, *p* = 0.40). The average reaction time and throughput score on the working memory task was 1795 ± 524 ms and 31 ± 11, respectively. The ΔHbDiff response was correlated with reaction time (r = 0.44, *P* = 0.03) and throughput score (r = − 0.57, *p* = 0.005) such that higher ΔHbDiff responses were associated with worse cognitive performance.

**Figure 2:**
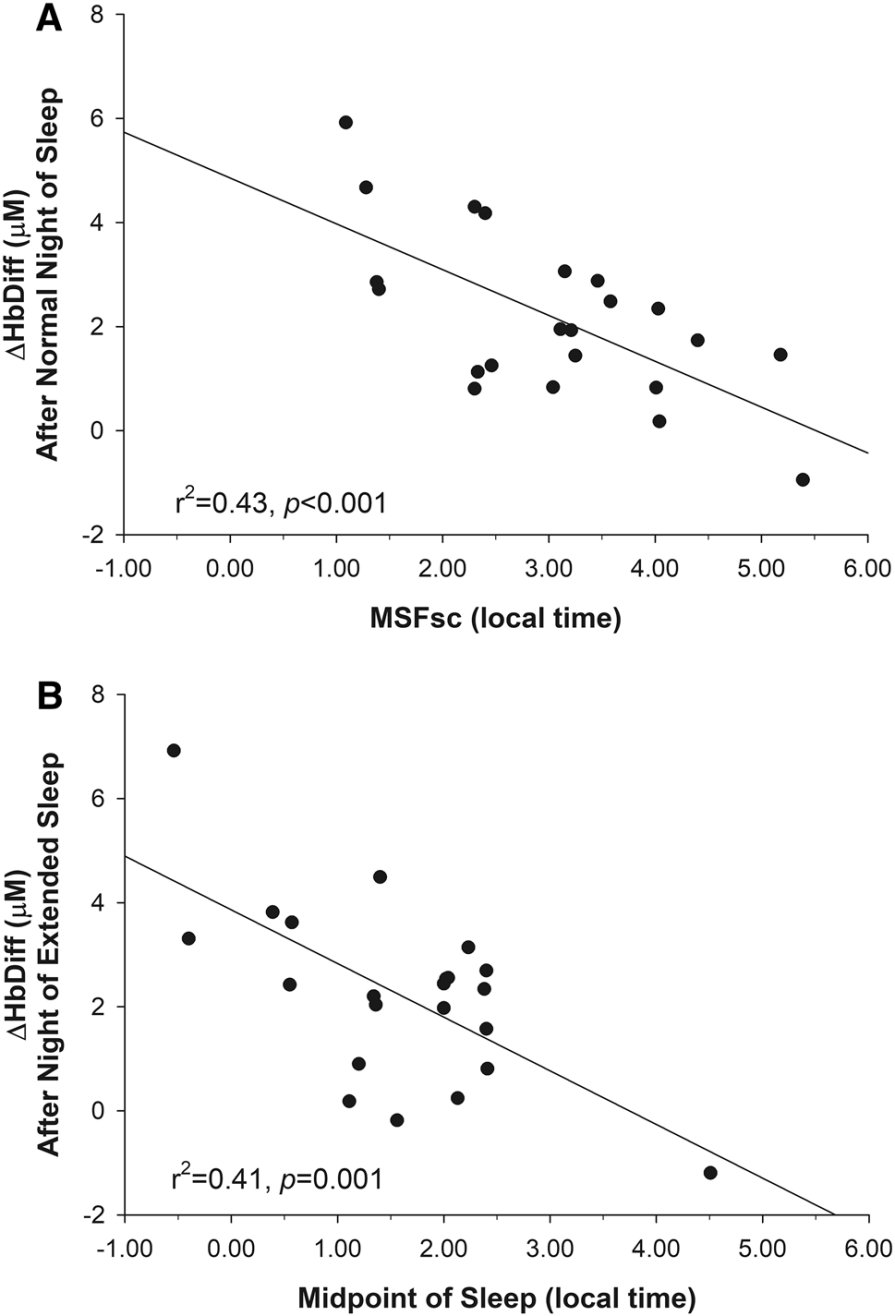
Relationship between regional cerebral oxygenation response (ΔHbDiff) to a visuospatial working memory task and (**A**) chronotype as assessed by the midpoint of sleep on free days corrected for sleep debt on workdays (MSFsc) as well as (**B**) the midpoint of sleep measured during the single night of sleep extension.

Table 1 presents the association between sleep parameters, cognitive performance, and prefrontal cerebral oxygenation. No sleep parameter was correlated with cognitive performance on the working memory task. As expected, habitual sleep duration was positively correlated with ΔHbDiff (r^2^ = 0.18, *p* = 0.04), along with time in bed (r^2^ = 0.33, *p* = 0.004), wake after sleep onset (r^2^ = 0.25, *p* = 0.01), sleep fragmentation index (r^2^ = 0.21, *p* = 0.02), and MSFsc (r^2^ = 0.43, *p* < 0.001). The relationship between ΔHbDiff and MSFsc was such that earlier chronotypes had higher cerebral oxygenation responses than later chronotypes (Fig. 2A). After controlling for variance explained by MSFsc using partial regression, only time in bed remained significantly correlated with ΔHbDiff (adjusted r^2^ = − 0.21, *p* = 0.02; Table 1). It should be noted that controlling for variance explained by sleep duration using partial regression did not change the significant correlation between ΔHbDiff and MSFsc (r = − 0.62, r^2^ = 0.38, *p* = 0.001).

**Table 1:** Correlation coefficients between sleep (12-night average) and prefrontal cerebral oxygenation.

	Reaction time	*p* value	Throughput score	*p* value	ΔHbDiff	*p* value	ΔHbDiff (adj.*)	*p* value
Time in bed	0.31	0.15	− 0.36	0.09	0.58	0.004	0.46	0.02
Sleep duration	0.27	0.21	− 0.25	0.25	0.43	0.04	0.34	0.11
Wake after sleep onset	0.19	0.39	− 0.36	0.09	0.50	0.01	0.37	0.08
Awakenings	0.15	0.49	− 0.22	0.32	0.22	0.31	0.15	0.48
Sleep efficiency	− 0.10	0.64	0.28	0.20	− 0.31	0.14	− 0.19	0.37
Fragmentation index	0.24	0.28	− 0.31	0.16	0.46	0.02	0.31	0.14
Sleepiness rating	− 0.05	0.80	− 0.18	0.40	− 0.01	0.96	0.07	0.75
Social jet lag	− 0.33	0.11	0.25	0.24	− 0.02	0.89	0.13	0.54
MSFsc	− 0.21	0.34	0.26	0.23	− 0.66	< 0.001		

ΔHbDiff, prefrontal cerebral oxygenation response to working memory task; *, adjusted for MSF_SC_ (chronotype).

### Effect of a single night of sleep extension

Table 2 shows that a single night of sleep extension increased (*p* < 0.01) time in bed, sleep duration, wake after sleep onset, and nocturnal awakenings as compared to habitual sleep. The midpoint of sleep during extension was shifted to an earlier local time (~ 70 min earlier) indicating that participants extended sleep by going to bed earlier rather than sleeping later as per study design. There was no difference in ΔHbDiff (extended vs. habitual: 2.21 ± 1.74 vs. 2.18 ± 1.59 μM, *p* = 0.86, Hedges’ *g* = 0.01), reaction time (1842 ± 551 vs. 1795 ± 524 ms, *p* = 0.27, Hedges’ *g* = 0.08), or throughput scores (31 ± 10 vs. 31 ± 11, *p* = 0.68, Hedges’ *g* = 0.00) following sleep extension as compared to after the night of normal sleep. The ΔHbDiff response measured after sleep extension was correlated with MSFsc (r = − 0.63, *p* = 0.001) and the midpoint of sleep during the night of extended sleep (r = − 0.64, *p* = 0.001; Fig. 2B).

**Table 2:** Comparison of sleep parameters after one night of extended sleep.

	12-night average	Sleep extension	*p* value	Hedges’ *g*
Time in bed (min)	469 ± 51	633 ± 41	< 0.001	3.54
Sleep duration (min)	407 ± 45	549 ± 36	< 0.001	3.48
Wake after sleep onset (min)	59 ± 19	78 ± 40	0.007	0.60
Awakenings (#)	21 ± 5	25 ± 9	0.001	0.54
Sleep efficiency (%)	86 ± 3	87 ± 5	0.29	0.24
Sleep fragmentation index (%)	23 ± 8	21 ± 10	0.27	0.22
Sleepiness rating	5.9 ± 3.2			
Social jet lag (min)	23 ± 50			
Midpoint of sleep (median local time)	3:13 a.m. (MSFsc)	2:01 a.m		

Values are mean ± SD unless otherwise stated.

### Impact of chronotype on cerebral oxygenation following sleep extension

**Figure 3:**
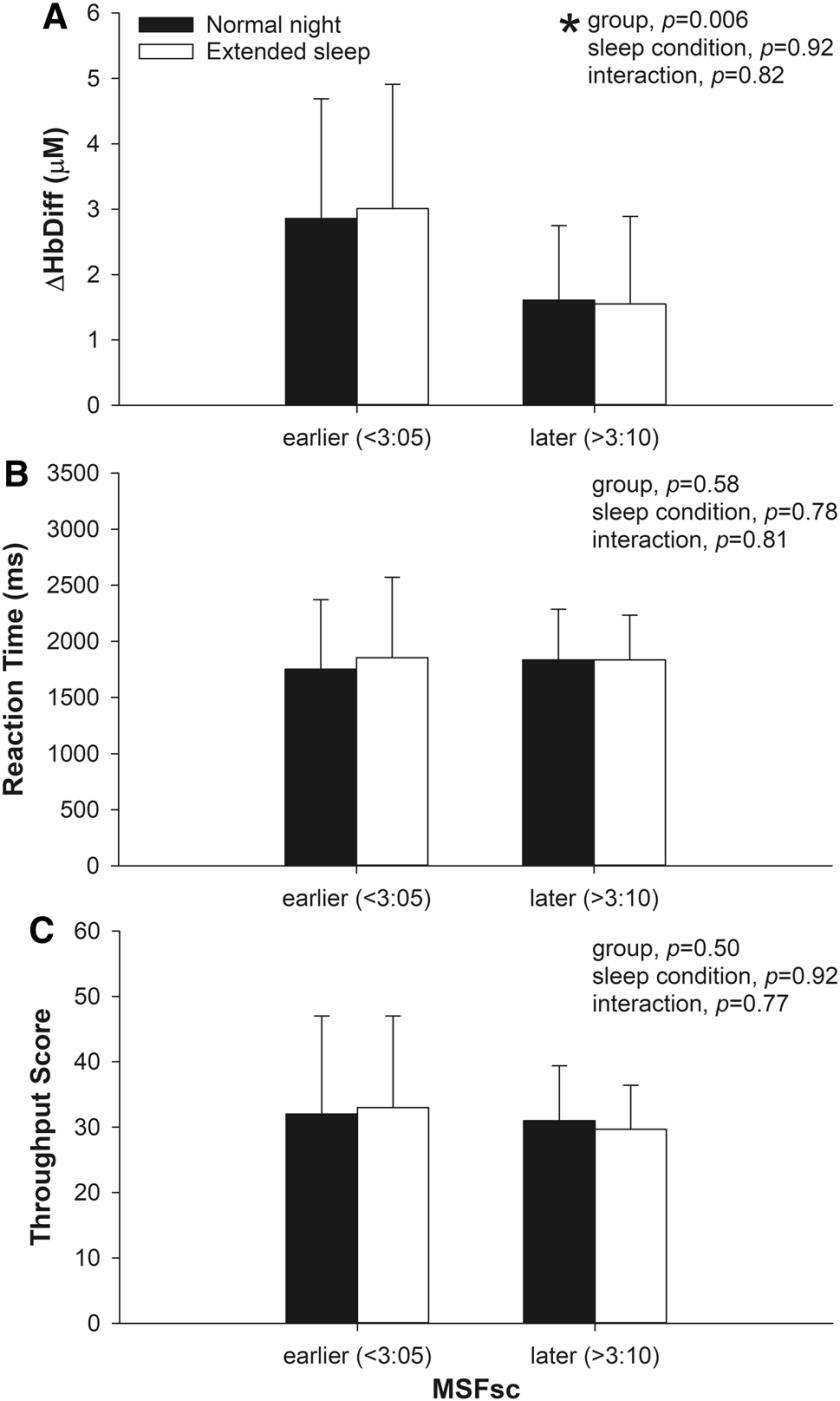
Comparison of regional cerebral oxygenation (ΔHbDiff), reaction time, and throughput score measured during a visuospatial working memory task between earlier and later chronotype groups stratified based on chronotype as assessed by the midpoint of sleep on free days corrected for sleep debt on workdays (MSFsc). Measurements were taken in the morning after a normal night of sleep or after a single night of sleep extension. *Main effect for group difference (*p* = 0.004).

To determine whether chronotype impacts prefrontal cerebral oxygenation changes following sleep extension, we divided participants into earlier (range 1:09–3:04 a.m., n = 10) and later (range 3:11–5:39 a.m., n = 12) chronotype groups using MSFsc. The shift to an earlier midpoint of sleep during sleep extension relative to MSFsc did not statistically differ between earlier (− 70 ± 27 min) and later (− 97 ± 52 min, *p* = 0.15, Hedges’ *g* = 0.63) chronotype groups. Both groups also had similar sleep duration during the night of extended sleep (earlier vs. later chronotype: 546 ± 24 vs. 552 ± 45 min, *p* = 0.72, Hedges’ *g* = 0.16). The ΔHbDiff response following sleep extension remained consistent with the ΔHbDiff response observed after the night of normal sleep with the earlier chronotypes showing higher ΔHbDiff than later chronotypes (mean difference = 1.35 μM, t = 2.87, *p* = 0.006, Hedges’ *g* = 0.89; Fig. 3A). Reaction time and throughput scores were similar within and between chronotype groups irrespective of sleep condition (Fig. 3B,C).

## Discussion

The objective of this study was to determine whether chronotype contributes to the association between longer sleep duration and higher regional cerebral oxygenation during cognitive functioning. We hypothesized that any relationship between sleep duration and cerebral oxygenation would be dependent on chronotype. In support of this hypothesis, we found that the correlation between longer habitual sleep duration and higher prefrontal cerebral oxygenation during a visuospatial working memory task was no longer statistically significant after controlling for variance explained by chronotype. Furthermore, we showed that increasing sleep duration through a single night of sleep extension does not alter prefrontal cerebral oxygenation. Interestingly, prefrontal cerebral oxygenation responses were tightly coupled to chronotype such that earlier chronotypes had higher prefrontal oxygenation responses than later chronotypes in the condition of normal sleep and after a single night of sleep extension despite longer sleep durations across all participants. Together, these observations highlight a close link between chronotype and brain activation responses to a cognitive challenge.

Several interesting insights can be taken from this study. First, sleep efficiency was not associated with prefrontal cerebral oxygenation during working memory processing which is consistent with past research in older adults using a verbal encoding task^23^. While sleep efficiency can be reduced by a greater number of nocturnal awakenings and wake after sleep onset, it does not seem to properly translate the negative effect of poor sleep quality on elevating brain activation as reported in young adults^23^. Second, social jet lag was unrelated to cognitive performance and prefrontal cerebral oxygenation responses in the present study indicating that misalignment between sleep timing on weekdays and the participants’ circadian phase was not an influential factor. Third, we did not find subjective ratings of sleepiness measured after the night of normal sleep to associate with cognitive performance or prefrontal cerebral oxygenation. Thus, the possibility that earlier chronotypes had higher prefrontal cerebral oxygenation as a result of functional compensation to maintain cognitive performance due to sleepiness is not supported by our results. This interpretation is consistent with previous studies that find subjective sleepiness and ability to sustain attention to be similar between earlier and later chronotypes when assessed in the morning^10,24^. Fourth, and lastly, our observation that longer sleep duration was correlated with higher prefrontal cerebral oxygenation responses during cognition is in agreement with previous studies^5,6^. However, an exploratory analysis of our data reveals that the association between habitual sleep duration and ΔHbDiff was only significant in later chronotypes (r = 0.68, *p* = 0.01) while the association was absent in earlier chronotypes (r = 0.09, *p* = 0.79). This suggests that the strength of the relationship may differ between chronotype groups. This result, however, should be interpreted with caution due to the low sample size within each chronotype group which underpowers our ability to fully test this question.

The reason for earlier chronotypes having higher prefrontal cerebral oxygenation during the cognitive challenge is unclear from our results, but may relate to earlier chronotypes having early declines in melatonin in the morning^9^ that may contribute to greater activation of the prefrontal cortex during cognitive challenges. For example, Killgore et al.^25^ observed that morning reductions in salivary melatonin are associated with higher brain activation in the prefrontal cortex during working memory testing. Another possible mechanism relates to higher core body temperatures reported in earlier chronotypes during morning clock hours^9^. Thermal research finds that a moderate rise in core body temperature (~ 1 °C) has the potential to elevate prefrontal cerebral oxygenation (ΔHbDiff) responses to cognitive functioning testing without changing cognitive performance^26^. These postulations are intriguing as they suggest circadian-related differences in physiological output between chronotype groups may influence brain function during cognition.

Several factors were not measured in this study that may have played a role in our results. Task difficulty increases frontal brain activation during working memory processing^1^. Despite familiarizing all participants to the cognitive test, we did not assess their impression of task complexity. Reaction time was correlated with ΔHbDiff such that longer response times were associated with higher prefrontal cerebral oxygenation responses as one may expect for a difficult task. Using reaction time as a surrogate for task difficulty and partial regression to control for its variance, we observed the relationship between MSFsc and ΔHbDiff remained significant (adjusted r = − 0.64, *p* = 0.001). So, we do not believe task difficulty explains our results. Another factor to consider is our regional assessment of brain activation. We were restricted by using NIRS to a depth-limited (~ 17.5 mm) area in the prefrontal cortex. Therefore, we cannot discern whether participants with higher prefrontal cerebral oxygenation responses were compensating for reductions in neural activity in other areas of the brain similar to what has been observed during matching to sample tasks following sleep deprivation^17^. Future research examining functional connectivity between brain networks is needed to further explore the influence of chronotype on brain activation.

Several study weaknesses should be mentioned. We examined apparently healthy middle-aged to older adults. Our rationale for examining middle-aged to older adults is that during this age range chronotype is advancing to earlier clock times from maximum ‘lateness’ reached in young adulthood^8^. This helped to ensure that this study captured a broad range of chronotypes for this investigation. We consider our sample healthy without sleep disorders, but we screened for signs of disordered sleep using a minimal number of self-report tools rather than objective methods like polysomnography. Therefore, it is possible that we did not capture the presence of sleep disorders which are known to alter brain activation patterns during cognitive challenges^27^. Additionally, our sample size is relatively small, however, we were able to replicate results from the literature^5,6^; specifically, that sleep duration positively correlates with prefrontal cerebral oxygenation to test our hypothesis. Another limitation of this study is that we did not use the Morningness-Eveningness Questionnaire to characterize people into morning and evening types. Rather, we divided participants based on visual inspection of MSFsc, a proxy measurement for chronotype that is strongly correlated with delayed dim light melatonin onset^15^. Our approach produced two chronotype groups with average MSF_SC_ of 2:12 and 4:03 a.m., which is consistent with research that finds the circadian phase of evening types is delayed about 2-h compared to morning types^9,28^. In addition, the MSFsc cutoff employed in the present study to categorize the later chronotype group (> 3:10 a.m.) has been used recently to identify late chronotypes among a large cohort of middle-aged adults^29^. Lastly, we did not include a sleep restriction condition in this study to determine whether chronotype also explains variance in the rise in prefrontal activation after sleep loss as reported by others^1,2^, which makes a direct comparison a challenge.

In summary, chronotype is a sleep characteristic that explains much variance in working memory-dependent prefrontal cerebral oxygenation. This finding has significant implications for future research as it suggests that chronotype should be accounted for when assessing prefrontal brain activation patterns during cognitive functioning at a set time of day (e.g., morning clock times). Additionally, research examining the effect of altered sleep durations (e.g., sleep restriction and/or extension) should measure chronotype to determine whether observed changes in brain activation, if any, can be explained by an individual’s endogenous circadian clock. Our study is timely as more attention is being given to circadian medicine to help improve human health^30^.

## Data Availability

The datasets used and/or analyzed during the current study are available from the corresponding author on reasonable request.

## References

[1] Chee MW, Choo WC (2004). Functional imaging of working memory after 24 hr of total sleep deprivation. J. Neurosci..

[2] Drummond SP, Gillin JC, Brown GG (2001). Increased cerebral response during a divided attention task following sleep deprivation. J. Sleep Res..

[3] Drummond SP, Brown GG (2001). The effects of total sleep deprivation on cerebral responses to cognitive performance. Neuropsychopharmacology.

[4] Soshi T (2010). Sleep deprivation influences diurnal variation of human time perception with prefrontal activity change: A functional near-infrared spectroscopy study. PLoS One.

[5] Suda M (2009). Subjective feeling of psychological fatigue is related to decreased reactivity in ventrolateral prefrontal cortex. Brain Res..

[6] Yeung MK, Lee TL, Cheung WK, Chan AS (2018). Frontal underactivation during working memory processing in adults with acute partial sleep deprivation: A near-infrared spectroscopy study. Front. Psychol..

[7] Clark C, Rivas E, Gonzales JU (2022). Six nights of sleep extension increases regional cerebral oxygenation without modifying cognitive performance at rest or following acute aerobic exercise. J. Sleep Res..

[8] Roenneberg T (2004). A marker for the end of adolescence. Curr. Biol..

[9] Duffy JF, Dijk DJ, Hall EF, Czeisler CA (1999). Relationship of endogenous circadian melatonin and temperature rhythms to self-reported preference for morning or evening activity in young and older people. J. Investig. Med..

[10] Taillard J, Philip P, Bioulac B (1999). Morningness/eveningness and the need for sleep. J. Sleep Res..

[11] Schmidt C (2015). Pushing the limits: Chronotype and time of day modulate working memory-dependent cerebral activity. Front. Neurol..

[12] Putilov AA, Poluektov MG, Dorokhov VB (2020). Evening chronotype, late weekend sleep times and social jetlag as possible causes of sleep curtailment after maintaining perennial DST: Ain't they as black as they are painted?. Chronobiol. Int..

[13] Okun ML (2009). Psychometric evaluation of the insomnia symptom questionnaire: A self-report measure to identify chronic insomnia. J. Clin. Sleep Med..

[14] Johns MW (1991). A new method for measuring daytime sleepiness: the Epworth sleepiness scale. Sleep.

[15] Kantermann T, Sung H, Burgess HJ (2015). Comparing the morningness-eveningness questionnaire and Munich chronotype questionnaire to the dim light melatonin onset. J. Biol. Rhythms.

[16] Kabat MH, Kane RL, Jefferson AL, DiPino RK (2001). Construct validity of selected automated neuropsychological assessment metrics (ANAM) battery measures. Clin. Neuropsychol..

[17] Habeck C (2004). An event-related fMRI study of the neurobehavioral impact of sleep deprivation on performance of a delayed-match-to-sample task. Brain Res. Cogn. Brain Res..

[18] Smith ME, McEvoy LK, Gevins A (2002). The impact of moderate sleep loss on neurophysiologic signals during working-memory task performance. Sleep.

[19] Daniel TA, Katz JS, Robinson JL (2016). Delayed match-to-sample in working memory: A BrainMap meta-analysis. Biol. Psychol..

[20] Rice, V. J., Alfred, P. E., Boykin, G. L., DeVilbiss, C. & Bateman, R. *Automated Neuropsychological Assessment Metrics (ANAM) Traumatic Brain Injury (TBI): Human Factors Assessment* 1–42 (2011). https://apps.dtic.mil/sti/pdfs/ADA549141.pdf

[21] Duncan A (1996). Measurement of cranial optical path length as a function of age using phase resolved near infrared spectroscopy. Pediatr. Res..

[22] Colier WN, van Haaren NJ, Oeseburg B (1995). A comparative study of two near infrared spectrophotometers for the assessment of cerebral haemodynamics. Acta Anaesthesiol. Scand. Suppl..

[23] Jonelis MB (2012). Age-related influences of prior sleep on brain activation during verbal encoding. Front. Neurol..

[24] Schmidt C (2009). Homeostatic sleep pressure and responses to sustained attention in the suprachiasmatic area. Science.

[25] Killgore WDS, Kent HC, Knight SA, Alkozei A (2018). Changes in morning salivary melatonin correlate with prefrontal responses during working memory performance. Neuroreport.

[26] Ashworth ET, Cotter JD, Kilding AE (2021). Impact of elevated core temperature on cognition in hot environments within a military context. Eur. J. Appl. Physiol..

[27] Ayalon L, Ancoli-Israel S, Klemfuss Z, Shalauta MD, Drummond SP (2006). Increased brain activation during verbal learning in obstructive sleep apnea. Neuroimage.

[28] Taillard J (2011). Time course of neurobehavioral alertness during extended wakefulness in morning- and evening-type healthy sleepers. Chronobiol. Int..

[29] Kim HJ (2023). Earlier chronotype in midlife as a predictor of accelerated brain aging: A population-based longitudinal cohort study. Sleep.

[30] Kramer A (2023). Time for circadian medicine. Acta Physiol. (Oxf.).

